# Surgical Diseases

**Published:** 1876-04

**Authors:** 


### SURGICAL DISEASES.

### EXTERNAL PILES

are tumors at the edge of the anus, covered with skin and mucous membrane; they are external to the muscle which closes the anus, and are called

### BLIND PILES, because they do not bleed. Whatever interferes with the circulation of the blood about the parts causes piles; fullness and tension of the abdominal vessels does the same thing. The most common cause of piles is constipation, liver diseases, and pregnancy; at least these used to be the most common causes, but of late years the ailment has been most frightfully aggravated in character, and increased in numbers, by the indiscreet use of aperient and purgative medicines, whether in the shape of pills, or waters from celebrated springs, or other liquids.

Worms are also a cause of piles. Piles may be indolent or inflamed. When indolent, their inconvenience results from their size and situation, and the pain arising from their coming within the grip of the sphincter muscle, that which closes the lower bowel after defecation. When inflamed they occasion pain, heat, itching, fullness, and a feeling of tension, a feeling as if there was something to come away, and yet it can not be made to do so. These and other symptoms may be complicated with inflammation of the bladder, pain in the thighs, back, etc.; sometimes it is an aching feeling, and there are mucous discharges. The skillful surgeon feels himself perfectly at home in all these ailments, and affords speedy and most grateful relief and permanent and perfect cure, when of comparatively recent origin; but when they have been long neglected, and there are marked inflammatory symptoms, a cure is effected in one or two ways, either by applications to the surface or by ligature. Piles should not be cut or removed with the knife. The long continuance of piles is always destructive of health, and invariably shortens life, making it meanwhile, in a great many cases, a torture and a burden.

WARTS, and other excrescences about the anus, are usually removed by external applications.

### Fistula in Ano

Is an ulcerated canal in the neighborhood of the anus, and is the result of an abscess or injury to the parts implicated. The fistula gives rise to soreness, is continually discharging blood and matter, and never gets well of itself, because the muscles of the parts are too incessantly in action to permit any healing process, and besides, fœcal matter is constantly passing over the sore. All palliative treatment further than perfect cleanliness is worse than useless, because valuable time is lost. Drugs injure the general health without having the slightest possible good effect on the fistula itself. The continued existence of a fistula in these parts always undermines the constitution, destroys all mental and moral comfort, and finally life itself. The usual remedy is the knife. It can be cured without this violence, and more certainly. Cases are on record where the evil remained after fifteen and even twenty-one cuttings with the knife. The other method is perfectly safe, always efficient, and without suffering.

### Anal Fissure

And excoriations thereabouts cause the most intolerable sufferings during evacuations; there is often intense and constant itching. It requires the most practiced eye to detect the immediate nature and causes of these affections; but when determined, the appropriate external applications effectually and speedily remove the trouble. Last year, a citizen called, whc had literally scratched holes in his flesh in his endeavor to relieve intolerable itching. He had been taking and using every thing he could hear of as even likely to avail, without the slightest given effect. He was cured within a fortnight.

### Prolaplus Ani

Is a reversion of the lower bowel and its protrusion, very much as in the pulling of a purse, or rather, the lining of a purse inside out. This is caused by a defect in the structure itself, or through violent strainings, as a result of piles, stricture, etc.

"Nelaton's Suppository" gives immediate relief, and is the most effectual remedy to prevent a return of the malady. It will usually cure costiveness, which is the prime cause of all these troubles and dangers.

### Stricture of the Rectum

Is caused by a thickening of its coats and a consequent diminution of the size of the canal. As it may be accompanied by some malignant disease, professional advice should be promptly sought in all cases.

### Stricture of the Urethra

Is the contraction caused by inflammation. The commencement of stricture gives rise commonly to the following symptoms — a frequent desire to urinate, accompanied with uneasy sensations; a few drops remain after urination, and subsequently dribble away; the stream of water is smaller than usual, and is forked, scattered, or twisted; longer time and greater efforts are required to pass water; itching and gleety discharges are occasional concomitants. The inroads of stricture are most insidious. Sometimes for years the patient may not feel any special uneasiness, but at length the urethra diameter becomes so small as scarcely to allow the passage of any urine except in drops, and even that by hard straining. Ultimately the most disastrous consequences take place; fistulous openings occur externally, and the most severe surgical operations have to be submitted to, if life enough is left to encounter them. Bodenhamer records a case where several of these openings occurred on the inner portion of the thigh, and these were the only outlets for the urine, which came away in drops day and night. A most horrible state of things, and all the result of a want of a little discreet surgical attention timely given. And a main object of these pages is to induce persons to give a prompt and early attention to the first symptoms of any of these maladies.

